# The impact of earthquakes on the frequency and prognosis of the most common emergency cardiac conditions

**DOI:** 10.3325/cmj.2023.64.164

**Published:** 2023-06

**Authors:** Zdravko Babić, Marin Pavlov, Petra Radić, Josica Šikić, Edvard Galić, Diana Balenović, Tomislav Letilović, Davor Horvat, Luka Perčin, Dubravka Šipuš, Valentina Obadić, Davor Miličić

**Affiliations:** 1 Department of Cardiology, Sestre Milosrdnice University Hospital Center, Zagreb, Croatia; 2 Department of Cardiology Dubrava University Hospital, Zagreb, Croatia; 3 Department of Cardiology, Sveti Duh University Hospital, Zagreb, Croatia; 4 Department of Cardiology, Dr. Ivo Pedišić General Hospital, Sisak, Croatia; 5 Department of Cardiology, Merkur University Hospital, University of Zagreb, School of Medicine, Zagreb, Croatia; 6 Department of Cardiology, Karlovac General Hospital, Karlovac, Croatia; 7 Department of Cardiology, University Hospital Center Zagreb, Zagreb, Croatia; 8 Department of Cardiology, Merkur University Hospital, University of Zagreb, School of Medicine, Zagreb, Croatia

## Abstract

**Aim:**

To assess whether the number of patients with a cardiac chief complaint and their characteristics differed between before and after two major earthquakes that struck Croatia in 2020.

**Methods:**

We collected data on all visits of patients with a cardiac chief complaint examined in the emergency departments of six hospitals nearest to the epicenters. Patients seen during the 7 days before the earthquake were compared with those seen on the day and during the 6 days after the earthquake.

**Results:**

Patients seen after the earthquake were younger (68 [59-79] vs 72.5 [65-80]; *P* < 0.001) and less frequently had cardiovascular disease (32.9% vs 42.8%; *P* < 0.001). This group less frequently had the primary diagnosis of acute myocardial infarction (AMI) (15.6% vs 21.9%; *P* = 0.005), heart failure (9.3% vs 19.4%; *P* < 0.001), dysregulated hypertension (13.9% vs 19.4%; *P* = 0.01), but more frequently had non-anginal chest discomfort (28.8% vs 18.0%; *P* < 0.001). In a subgroup analysis of patients seen in hospitals located within 20 km from the epicenter, significantly more patients seen after the earthquake compared with those seen before the earthquake presented with AMI (14.5% vs 22.8%; *P* = 0.028), acute elevation of blood pressure (10% vs 21.8%, *P* = 0.001), and paroxysmal arrhythmias treated with electrocardioversion (0.9% vs 4.5%, *P* = 0.022).

**Conclusion:**

After two moderately strong earthquakes, hospitals within 20 km from the epicenter saw a significant increase in acute cardiac conditions such as elevated blood pressure, AMI, and cardioverted arrhythmias. Eventually, these earthquakes had no impact on the outcomes of the studied population.

A natural disaster is an event with a negative impact following a natural hazard such as an avalanche, flooding, drought, or earthquake (1-3). Natural disasters lead to an increased incidence of communicable diseases, non-communicable diseases, and trauma (4-7). Among all non-communicable diseases, cardiovascular diseases remain the main disease category related to mortality in the contemporary population (8-10).

Earthquakes are associated with the occurrence of acute cardiovascular syndromes. A review of 26 studies on 12 earthquakes (11) found nine events to be related to a significantly higher incidence of acute coronary syndromes (ACS).

This study focused on two earthquakes that struck Croatia in 2020. On March 22, a 5.5 earthquake on the Richter scale hit Zagreb, the capital of Croatia. One person died, 27 people were injured, and major material damage occurred in the historic city center (12). Six months later, on December 29, a 6.2 magnitude earthquake occurred near Petrinja (a town 58 km from Zagreb; 45.4002°N, 16.2187°E). Hundreds of after-shocks with a magnitude greater than 1.0 degrees Richter ensued. The earthquake killed seven and injured several dozens of people (12), destroying many buildings and causing catastrophic material damage.

We sought to assess whether the number of patients with a cardiac chief complaint and their characteristics changed between before and after the earthquake. We also conducted a subanalysis only in hospitals located less than 20 km from the epicenter.

## Patients and methods

We retrospectively collected data on all emergency visits of patients with a cardiac chief complaint in the emergency departments (ED) of the six hospitals closest to the epicenters: Sestre Milosrdnice University Hospital Center (UHC), Zagreb UHC, Sveti Duh University Hospital (UH), Merkur UH (all in Zagreb), Ivo Pedišić General Hospital (Sisak), and Karlovac General Hospital (Karlovac).

Data on patient characteristics, emergency and in-hospital work-up, admission, main diagnosis, hospital stay, and outcome were collected from digital medical records. The data were collected for the 7 days before the earthquakes, for the day of each earthquake, and for the 6 days after the earthquakes. Patients were divided into two groups: those seen before the earthquake and those seen on the day or after the earthquake. In addition, a subgroup analysis included only the hospitals located within 20 km from each epicenter.

### Statistical analysis

The normality of distribution was tested with a Kolmogorov-Smirnov test. Descriptive variables are presented as means and standard deviations (SD) or medians and interquartile ranges (IQR). Categorical variables are presented as counts and frequencies. The differences between categorical variables were assessed with a χ^2^ test and those between continuous variables with a Mann-Whitney U test. The significance tests were two-tailed, and *P* < 0.05 was considered significant. Statistical analyses were performed with SPSS for Windows, version 25 (IBM, Armonk, NY, USA).

## Results

In the studied period, there were 5575 ED visits, of which 1251 (22.4%) were with a cardiac chief complaint. The distances from the hospitals to the epicenters and the number of patients examined in each hospital are presented in Table 1. In the entire sample with cardiac chief complaint, there was a predominance of male patients (55.2%). The median age was 67 (53-78) years (Table 2).

**Table 1:** Distance of the hospital from the epicenter and the number of patients examined in each hospital before and after the Zagreb earthquake of March 22 and the Petrinja earthquake of December 29, 2020

	Zagreb earthquake				Petrinja earthquake			
Hospital	distance from epicenter (km)	visits	cardiac	cardiac (%)	distance from epicenter (km)	visits	cardiac	cardiac (%)
Sestre Milosrdnice	7.173	322	136	42.2	50.545	731	213	29.1
Zagreb	3.464	741	112	15.1	49.855	1196	314	26.3
Sveti Duh	7.799	425	26	6.1	51.497	724	33	4.
Merkur	4.113	185	37	20.0	49.791	460	67	14.6%
Ivo Pedišić (Sisak)	49.121	308	117	38.0	14.465	323	112	34.7
Karlovac	56.285	70	28	40.0	53.745	90	56	62.2

**Table 2:** Comparison of patients examined in the emergency department (ED) before the earthquake (EQ) and on the day and after the EQ

	Whole sample			Within 20 km from epicenter		
	before EQ	on the day or after EQ	p	before EQ	on the day or after EQ	*P*
	count (%) or median (interquartile range)			count (%) or median (interquartile range)		
Age	72 (65-80)	68 (59-79)	<0.001	71 (59-80)	66 (57-76)	0.004
Female sex	283 (46.9)	277 (42.9)	0.158	108 (48.9)	76 (37.6)	0.020
Medical history:						
hypertension	475 (78.9)	407 (63.4)	<0.001	182 (82.4)	147 (72.8)	0.018
dyslipidemia	247 (41.2)	210 (32.9)	0.002	97 (43.9)	87 (43.1)	0.865
diabetes	120 (20.0)	109 (17.1)	0.182	48 (21.8)	44 (21.9)	0.986
active smoking	125 (23.4)	148 (25.9)	0.333	46 (23.0)	58 (32.6)	0.037
previous coronary artery disease	258 (42.8)	210 (32.9)	<0.001	110 (49.8)	78 (38.6)	0.021
Admission	251 (41.7)	223 (34.6)	0.010	89 (40.5)	83 (41.1)	0.895
Time in ED	4 (2-7.25)	4 (1.5-8)	0.066	4 (2-6)	4 (2.15-8)	0.099
Hospital stay	5 (3-9)	5 (3-7)	0.195	4 (2-6)	4 (2-7)	0.448
Non-anginal chest discomfort	109 (18.0)	186 (28.8)	<0.001	27 (12.2)	19. (9.4)	0.354
Main diagnosis						
myocardial infarction	100 (16.6)	89 (13.8)	0.170	32 (14.5)	46 (22.8)	0.028
unstable angina	32 (5.3)	12 (1.9)	0.001	14 (6.3)	5 (2.5)	0.056
decompensated heart failure	117 (19.4)	60 (9.3)	<0.001	53 (24.0)	22 (10.9)	<0.001
arrhythmia	114 (18.9)	95 (14.7)	0.048	47 (21.3)	34 (16.8)	0.247
hypertension	117 (19.4)	90 (13.9)	0.010	22 (10.0)	44 (21.8)	0.001
Coronary angiography only	5 (0.8)	9 (1.4)	0.341	1 (0.5)	2 (1.0)	0.510
Percutaneous coronary intervention	80 (13.2)	74 (11.5)	0.341	28 (12.7)	35 (17.3)	0.179
Electrical cardioversion	10 (1.7)	13 (2.0)	0.636	2 (0.9)	9 (4.5)	0.022
Mechanical ventilation	12 (2.0)	9 (1.4)	0.417	5 (2.3)	2 (1.0)	0.306
Cardiopulmonary resuscitation	11 (1.8)	9 (1.4)	0.549	4 (1.8)	3 (1.5)	0.794
In-hospital mortality	19 (3.2)	13 (2.0)	0.201	6 (2.7)	5 (2.5)	0.877

The average admittance rate was 38.0%. The median time spent in the ED was 4 h (1.5-8 h). The median hospital stay was 5 days (3-8 days). Patients seen after the earthquake were younger (68 [59-79] vs 72.5 [65-80]; *P* < 0.001) and less frequently had cardiovascular disease (32.9% vs 42.8%; *P* < 0.001). This group less frequently had the primary diagnosis of acute myocardial infarction (AMI) (15.6% vs 21.9%; *P* = 0.005), heart failure (9.3% vs 19.4%; *P* < 0.001), dysregulated hypertension (13.9% vs 19.4%; *P* = 0.01), but more frequently had non-anginal chest discomfort (28.8% vs 18.0%; *P* < 0.001) (Table 2).

In the subgroup analysis of patients seen in EDs located within 20 km from the epicenter, significantly more patients seen after the earthquake compared with those seen before the earthquake presented with AMI (14.5% vs 22.8%; *P* = 0.028), acute elevation of blood pressure (10% vs 21.8%, *P* = 0.001), and paroxysmal arrhythmias treated with electrocardioversion (0.9% vs 4.5%, *P* = 0.022) (Table 2). Previously detected differences in the occurrence of arrhythmias, unstable angina, and non-anginal chest discomfort were no longer significant (Table 2). The earthquake had no effect on the outcome of these patients.

## Discussion

This multicenter study found a higher incidence of acute AMI among patients examined on the day and after the earthquake compared with the days before the event. However, this was true only for hospitals located within 20 km from the epicenter.

An earthquake represents a major stressful event. After acute psychological stress, certain patients are hyperresponsive to the activation of the sympathetic nervous system (13). Hypersensitivity may cause coronary and vascular atherosclerotic disease to become acute due to autonomic dysfunction, inflammatory, neuroendocrine, and platelet activation, and the possibility of vulnerable plaque disruption. This pathophysiological mechanism was proposed by a study analyzing the time and frequency domain of heart rate variability in patients undergoing 24-hour Holter electrocardiography during the 1999 Taiwan earthquake (14). The same mechanism is also suggested by our data, given the significant increase in the number of patients with high arterial pressure and paroxysmal arrhythmias treated with electrocardioversion in hospitals within 20 km from the epicenter. Emotional stress may continue in the days following the earthquake owing to property damage, reduced employment opportunities, need for reconstruction, altered daily routines, and social disruption (15).

Our study highlighted the importance of the distance between the epicenter and the treating hospital for the incidence of myocardial infarction and other cardiovascular events. An increase in acute cardiovascular events was reported following the 1995 Hanshin-Awaji Earthquake (16). Cardiovascular mortality increased during the nighttime period, especially among elderly patients residing in the vicinity of the epicenter (16). One of the contributing factors to the increase in ACS mortality was the disruption and unavailability of medical services (17). In the week following the earthquake, the number of hospital admissions due to AMI increased, a result corresponding to our findings (18). The incidence of myocardial infarctions normalized after four weeks (19). A study on the January 1994 earthquake in Los Angeles, which included data from more than 100 hospitals, revealed an increase in AMIs from 149 a week before the earthquake to 201 in the week after the earthquake (20,21). The data were corroborated by the coroners' findings, which showed an increase in the number of sudden deaths due to cardiac causes, from an average of 4.6 per day before the earthquake to 24 on the day of the earthquake (20). After an earthquake in northeast Japan in 2011, the number of myocardial infarction cases peaked in the first week (22). The incidence of myocardial infarctions was positively correlated with the strength of the earthquake (22).

A Japanese study from 2014 (23) assessed the rate of reperfusion, time course of treatment, and intrahospital mortality of patients with ST elevation myocardial infarction before and after the catastrophic 2011 earthquake and tsunami in Iwate Prefecture. The rate of percutaneous coronary intervention (PCI) in the inland area not affected by the tsunami was equal pre- and post-disaster (around 80%), a finding that is in agreement with our data. However, in the tsunami-affected area, door-to-balloon time in 2011 was significantly shorter than in 2010, and the PCI rate was significantly lower. Intrahospital mortality tripled (23). Fortunately, in both earthquakes in Croatia, all elements of the primary PCI network functioned without interruption. Hence, no excess cardiovascular mortality was observed.

A study on two earthquakes in Christchurch, New Zealand (September 4, 2010 and February 22, 2011) showed a significant increase in cardiac-related hospital admissions, ST elevation myocardial infarctions, and non-cardiac chest pain in the first two weeks after the September earthquake (magnitude 7.1) but not after the February earthquake (magnitude 6.3) (24). This suggests the importance of the earthquake magnitude on clinical events following the disaster.

Research on the Noto Peninsula in Japan (25) showed that an increase in ACS after an earthquake can be found in rural areas as well. Most of the ACS cases occurred within 7 days after the earthquake.

In patients without coronary heart disease, severe psychophysical stress can lead to stress-induced heart failure and Takotsubo cardiomyopathy (TC) (15). We observed a decrease in decompensated heart failure regardless of the distance from the epicenter. The fact that fewer patients with heart failure symptoms, unlike patients with AMI, visited the ED after the earthquake could be explained by the differences in the pathophysiology and dynamics of the two cardiac conditions. An increase in the incidence of TC, which could correspond to stress-induced heart failure, was observed after the mid-Niigata Prefecture earthquake (26). We found no increase in the occurrence of TC.

Most studies involved in the systematic review by Bazoukis et al (11) reported a short-term effect of earthquakes on the incidence of myocardial infarction. Systolic blood pressure measured by patients at home was significantly elevated for two weeks after the earthquake, while there was no difference after four weeks (11). The same was observed for heart rate values. Furthermore, cardiac-related hospital admissions significantly increased in the first two weeks following the earthquake (11). This indicates that stressful events such as earthquakes have clear short-term health-related consequences, which may lead to an increased need for cardiological treatment and care.

Health systems, especially those in regions with elevated cardiovascular risks, should be prepared for the treatment of cardiovascular diseases after natural disasters. Emergency services should be ready to provide assistance not only to the injured but also to an increased number of patients with cardiovascular diseases. Additionally, the general population should be educated to administer first aid and cardiopulmonary resuscitation, measures that can lead to improved outcomes in patients who develop an acute cardiovascular event. The effects of a devastating event can be long lasting, and, in addition to medical help, psychological help is often needed.

There are several limitations to this study. First, this was a retrospective study; however, it is almost impossible to investigate an unpredictable event such as an earthquake in a prospective manner. Second, considering that Sisak General Hospital sustained substantial material damage during the second earthquake, an unknown number of patients may not have reached the hospital emergency service. Third, the earthquakes were felt in a much wider area than the one investigated, hence, some potential influences could have been omitted.

In conclusion, the prevalence of CVDs is increased after an earthquake. Similar to previous studies, we found an increase in some cardiac emergencies following earthquake events. After the two moderately strong earthquakes investigated in this study, there were more patients with precordial oppressions, who were younger and had fewer cardiovascular risk factors. On the other hand, in hospitals within 20 km from the epicenters, we found a significant increase in acute cardiac conditions, such as elevated blood pressure, AMI, and cardioverted arrhythmias. Eventually, these earthquakes had no impact on the outcomes of the studied population.
